# The pathogen *Mycoplasma dispar* Shows High Minimum Inhibitory Concentrations for Antimicrobials Commonly Used for Bovine Respiratory Disease

**DOI:** 10.3390/antibiotics9080460

**Published:** 2020-07-29

**Authors:** Marco Bottinelli, Marianna Merenda, Michele Gastaldelli, Micaela Picchi, Elisabetta Stefani, Robin A. J. Nicholas, Salvatore Catania

**Affiliations:** 1 Istituto Zooprofilattico Sperimentale delle Venezie, SCT1-Verona, 37135 Verona, Italy; mbottinelli@izsvenezie.it (M.B.); mmerenda@izsvenezie.it (M.M.); mgastaldelli@izsvenezie.it (M.G.); mpicchi@izsvenezie.it (M.P.); estefani@izsvenezie.it (E.S.); scatania@izsvenezie.it (S.C.); 2 The Oaks, Nutshell Lane, Upper Hale, Farnham, Surrey GU9 0HG, UK

**Keywords:** *Mycoplasma dispar*, minimum inhibitory concentration, antibiotic resistance, bovine respiratory disease

## Abstract

*Mycoplasma dispar* is an overlooked pathogen often involved in bovine respiratory disease (BRD), which affects cattle around the world. BRD results in lost production and high treatment and prevention costs. Additionally, chronic therapies with multiple antimicrobials may lead to antimicrobial resistance. Data on antimicrobial susceptibility to *M. dispar* is limited so minimum inhibitory concentrations (MIC) of a range of antimicrobials routinely used in BRD were evaluated using a broth microdilution technique for 41 *M. dispar* isolates collected in Italy between 2011–2019. While all isolates had low MIC values for florfenicol (<1 μg/mL), many showed high MIC values for erythromycin (MIC90 ≥8 μg/mL). Tilmicosin MIC values were higher (MIC50 = 32 μg/mL) than those for tylosin (MIC50 = 0.25 μg/mL). Seven isolates had high MIC values for lincomycin, tilmicosin and tylosin (≥32 μg/mL). More, alarmingly, results showed more than half the strains had high MICs for enrofloxacin, a member of the fluoroquinolone class considered critically important in human health. A time-dependent progressive drift of enrofloxacin MICs towards high-concentration values was observed, indicative of an on-going selection process among the isolates.

## 1. Introduction

*Mycoplasma dispar*, characterized for the first time in 1970 [1], is a mollicute frequently isolated from the respiratory airways of both healthy and pneumonic calves worldwide [2,3,4,5]. Phylogenetically, *M. dispar* falls within the *M. neurolyticum* cluster of the hominis group of *Mollicutes*, showing a high degree of similarity to *M. hyopneumoniae,* the cause of enzootic pneumonia in pigs worldwide, and *M. ovipneumoniae* [6], a respiratory pathogen of small ruminants. 

Few detailed studies have been carried out to establish the exact role of *M. dispar* in bovine respiratory disease (BRD) which is a chronic condition affecting beef cattle in feedlots and dairy calves; it is estimated to cost the USA cattle industry alone over US$4 billion in production losses, treatment and prevention [7]. Experimental infection of calves with *M. dispar* led to a mild pneumonia [8] but data from the field suggests a more pathogenic role. In the Netherlands *M. dispar* was isolated from 90% of pneumonic calves and only 40% of healthy lungs [3,4] while in Denmark it was found in over half of calf lungs showing either fibrino-necrotising or suppurative bronchopneumonia [9]. In all cases other pathogenic bacteria such as *Histophilus somni, Pasteurella multocida* and *Mannheimia haemolytica* were also present. *M. dispar* is detected frequently from pneumonic calves in the UK, and was believed to be the cause of a severe pleuropneumonia similar to the OIE listed contagious bovine pleuropneumonia in adult cattle which resulted in several deaths [5]. More evidence of its role in BRD has been described in North America where an initiating role for *M. dispar* leading to the invasion of *P. multocida* was proposed [10,11]. 

The pathogenicity mechanisms have been identified in *M. dispar* and include its ability to produce hydrogen peroxide and biofilm, both well-known virulence factors. A dose-dependent cytotoxicity in response to *M. dispar* has also been observed [12]. In addition, in vitro studies have shown that *M. dispar* is able to colonize the epithelial cells of the respiratory tract [13] similar to that seen by *M. hyopneumoniae* [14] potentially impairing the clearance of bacteria [15]. Furthermore, *M. dispar* has been shown to be immunosuppressive in the host [16]. For these reasons *M. dispar* is included with *M. bovis* amongst the agents that cause or exacerbate BRD [5,6]. However, unlike *M. bovis* which has been thoroughly studied during the past years [17,18], *M. dispar* has been neglected partly because it is extremely fastidious to culture and requires a specific medium to enable growth. Moreover, its colonies do not show–especially during the early passages–the typical “fried-egg” appearance. In addition, it is easily overgrown by other apparently less significant *Mycoplasma* species such as *M. bovirhinis,* often present in the respiratory tract of calves [5,7].

Calves affected by BRD are usually treated with antimicrobials even though some of these are not effective against mycoplasmas [18]. Consequently, there is a real danger of the emergence of resistance as a result of chronic therapies with multiple antimicrobials, which is already observed for the other BRD bacterial pathogens [19]. Information on the susceptibility of *M. dispar* to antimicrobial compounds is very scarce and, moreover, outdated [20]. In contrast, the susceptibility of *M. bovis* has been more widely studied and significant in vitro resistance has been seen during the last two decades [20,21,22,23,24]. The objective of this work was to carry out MIC tests on isolates of *M. dispar* identified in Italy over the last decade which will help guide veterinarians to choose the most appropriate first-line antibiotic for treating BRD involving *M. dispar*. This work shows how repeated, untargeted antibiotic treatments has changed the susceptibility of *M. dispar* to antimicrobials.

## 2. Results

The results of the type strain, *M. dispar* 462/2, NCTC 10125, were consistent throughout the study indicating good reproducibility of the test. MIC results for all isolates showed concordance when tested in duplicate. The distribution of the MIC values of the tested antimicrobials is showed in Table 1. More than half of the isolates (58.5%) had MIC values for enrofloxacin of greater than 8 µg/mL, with 11 isolates (26.8%) and 2 isolates (4.9%) having MIC values of 16 µg/mL and >16 µg/mL respectively. MIC50 and MIC90 values for enrofloxacin were very high, being 8 µg/mL and 16 µg/mL respectively. 

An unimodal distribution of doxycycline MIC value was observed, with 18 isolates (43.9%) being inhibited by a concentration of 1 µg/mL, even though 2 isolates (4.9%) had doxycycline MIC values of 8 µg/mL and one isolate had a MIC value of 16 µg/mL. In contrast, oxytetracycline MIC values showed a bimodal distribution with 16 isolates (39%) inhibited by concentrations lower than 1 µg/mL and 12 isolates (26.3%) having MIC values ≥16 µg/mL. MIC90 value for oxytetracycline is much higher (>32 µg/mL) compared to that for doxycycline (4 µg/mL).

Thirty-nine isolates (95.1%) had MIC values greater than the highest concentration of erythromycin used on the test plate (8 µg/mL), whereas 2 isolates had a MIC equal to 0.5 µg/mL and 4 µg/mL respectively. An unimodal distribution of tylosin MIC values was observed, with 33 isolates (80.5%) showing MIC values between 0.03125 and 1 µg/mL (MIC50 = 0.25 µg/mL), while 8 isolates (19.5%) showing MIC values of ≥32 µg/mL. Seven of these isolates also had very high MIC values for lincomycin, spiramycin and tilmicosin. 

Tilmicosin MIC values were distributed unimodally and revealed that 23 isolates (56.1%) were not inhibited by concentrations of ≥32 µg/mL. MIC50 and MIC90 values of tilmicosin were the highest registered, together with those of enrofloxacin and erythromycin. Three-fourths of the isolates (75.6%) had MIC values for spiramycin between 0.5 µg/mL and 2 µg/mL, while the rest (24.4%) showed very high MIC values (≥8 µg/mL), creating a clear bimodal distribution. 

With most of the isolates (90.2%) showing MIC values for tiamulin between 0.25 and 2 µg/mL, this drug appeared to have an unimodal distribution. Lincomycin MIC values were distributed unimodally as well, with three-quarters of the isolates (75.6%) having a MIC value between 0.5 and 1 µg/mL; however, 7 isolates (17.1%) showed MIC values of >32 µg/mL. MIC90 of lincomycin (>32 µg/mL) was the highest recorded together with that of oxytetracycline, erythromycin, tylosin, tilmicosin and spiramycin. Most of the isolates (78%) had MIC values for florfenicol equal or lower to 0.5 µg/mL, while the rest were inhibited by a concentration of 1 µg/mL.

**Figure 1:**
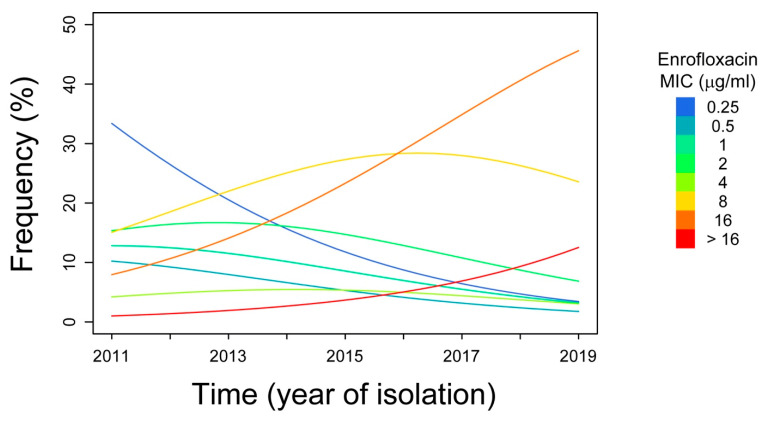
Variation in the frequency of MIC concentration of enrofloxacin over the period of study. The coloured lines indicate the trend of the different MIC value classes throughout the years.

A linear-by-linear association test on all microbials tested was performed to see whether there was a time-dependent variation in observation frequency of the different MIC values (see Table 2). The distribution of the isolates among the different values of MIC for enrofloxacin, tiamulin and tylosin varied over the study period. The Z-value sign (+/−) indicates the presence of a either a positive or a negative correlation between the ordinal variables. Apart from enrofloxacin, none of the antimicrobials showed a statistically significant positive correlation between time and MIC value classes. To substantiate this observation, enrofloxacin MIC data were analyzed using a proportional odds model which included the year of isolation (“time”) (Table 3). According to the model, with one unit increase in year of isolation, the log odds of observing increasing enrofloxacin MICs increased by 0.33 (95% confidence interval: 0.07–0.61), with a likelihood ratio test *p*-value of 0.0123. Accordingly, the estimated odd yearly increased by 1.39 times (95% confidence interval: 1.07–1.84). A progressive drift towards higher MIC value classes was clearly shown using model predictions of MIC-frequency profile between the years 2011–2019 (Figure 1).

## 3. Discussion

The antibiotic sensitivity of *M. bovis* and other BRD bacterial pathogens has been extensively studied, [18,19,22,25]. *M. dispar*, on the other hand, has been overlooked from previous surveys, probably because of its fastidiousness and/or the uncertainty of its pathogenic role; this has resulted in a consequent lack of interest from veterinary practitioners and researchers. The fact remains that *M. dispar*, which is widely prevalent in the bovine population, has been exposed to untargeted chemotherapy for many years. Therefore, we investigated whether there had been any changes in its in vitro susceptibility to antimicrobials particularly in view of the scarcity and outdated data available.

In this study, we found that more than half of the isolates showed MIC values for enrofloxacin greater than 8 µg/mL, a value considered to show poor effectiveness [26]. This is in stark contrast with data from previous studies from nearly 30 years ago showing MIC values for enrofloxacin well within the susceptible range [20]; here only a small number of isolates fell in this range. We also found a statistically significant, time-dependent progressive drift of enrofloxacin MICs towards high-concentration classes, indicative of an on-going selection process over time. It is interesting to note that, a similar phenomenon was recently observed in *M. synoviae,* a pathogen of poultry [27]. Taking into account the substantial differences between the two livestock sectors in terms of management and therapeutic treatments, we speculate that there is an analogous mechanism underlying the development of enrofloxacin resistance in these two *Mycoplasma* species. As proposed by Lysnyansky and collaborators [28], the widespread and probably incorrect use of fluoroquinolones is responsible for the emergence and dissemination enrofloxacin-resistant strains. In addition, a decreased susceptibility to enrofloxacin was recently seen for *M. bovis* in Italy [23], a mycoplasma that shares the same biological niche with *M. dispar*. It is highly likely then that the selective pressure exerted by the use of enrofloxacin on livestock has had the same effect on both *M. dispar* and *M. bovis*. This may have been facilitated by the transmission of resistance-genes between these two species, since the occurrence of horizontal gene transfer between phylogenetically distant *Mycoplasma* species has already been observed [29,30]. Moreover, the ability to acquire enrofloxacin resistance mutations from pre-existing resistant populations has been reported for *M. agalactiae,* a species phylogenetically very similar to *M. bovis* [31]. More studies are clearly needed to determine whether this occurs amongst mycoplasmas inhabiting the respiratory tract of cattle, but what is certain is that a more prudent use of enrofloxacin is necessary when treating animal mycoplasmoses.

Overall, the two tetracyclines tested in this study had different results. Oxytetracycline was less effective in vitro compared to doxycycline with more isolates falling into the intermediate susceptibility to resistant range confirming previous studies [20,32]. The *M. dispar* isolates appeared to behave similarly to Italian *M. bovis* isolates when exposed to oxytetracycline; in contrast, Barberio and collaborators reported higher MIC90s for doxycycline (8 µg/mL) with *M. bovis* isolates [33].

Most of the isolates in this study had MIC values greater than the highest concentration of erythromycin used in the test thus indicating a major lack of effectivity in vitro. Unfortunately, there are no previous published data on MIC value for erythromycin with *M. dispar* to make any comparison; however, many studies have shown that *M. bovis* isolates are resistant to this compound [33,34,35,36]. These data suggest that resistant mutants may have spread rapidly in the bovine population though little is known about the mechanisms behind the resistance against this 14-membered ring macrolide.

Regarding the 16-membered ring macrolides, in particular tylosin and spiramycin, we observed that our MIC50 values correspond to the MIC90 values reported in the literature [20]. Also, we noticed a bimodal distribution of MIC values for both antimicrobials in contrast to tilmicosin where a unimodal distribution with a distinct tendency towards the higher end of antibiotic concentrations. These data clearly show that *M. dispar* strains isolated over the last decade have poor susceptibility to macrolides. A similar situation was seen for Italian *M. bovis* isolates, which showed very high MIC50 and MIC90 values for these macrolides, although *M. bovis* resistance towards tylosin is higher than with *M. dispar* [23,33]. Again, sharing the biological niche and, consequently, being exposed to the same antimicrobials could have led to the same outcome for both these bovine *Mycoplasma* species. This is not surprising since macrolides are widely used as first-line antibiotic for treating BRD. 

Even though tiamulin is not licensed for cattle in Italy, it was included in this study to investigate its efficacy against *M. dispar* as a potential targeted treatment. Noteworthy, most of the isolates had low MIC values for tiamulin between 0.25 and 2 µg/mL, arranged unimodally, but overall had slightly higher values than those in previous studies [20,37] as well as for the pig pathogens *M. hyorhinis* [38] and *M. hyopneumoniae* [39] for which the antibiotic is licensed. These low values for *M. dispar* and, incidentally, *M. bovis* [33] may reflect the absence of exposure of these mycoplasmas to tiamulin which is not used for treating cattle. It would be interesting to investigate the discrepancies in the effectiveness of tilmicosin for swine and bovine mycoplasmas, especially since *M. dispar* and *M. hyopneumoniae* are phylogenetically very close to each other. 

As for erythromycin, there are no previous published data on MIC values for florfenicol for *M. dispar* to make any comparison. Most of our isolates were susceptible to florfenicol and future studies will use lower concentrations of the antibiotic to determine its full range of effectiveness. Surprisingly we did not find any similarity with MIC values of florfenicol for *M. bovis* which is reported to be less susceptible than *M. dispar* [23].

There was some evidence that lincomycin MIC values in this study were slightly higher than previous studies although over 75% of the present isolates had low MIC values. However, of concern, were smaller group of isolates with very high MICs strongly indicating in vitro resistance. Interestingly, these seven isolates also showed the highest MIC values for tylosin, spiramycin and tilmicosin. It is known that lincosamide and macrolide, although chemically distinct, act in a similar way inside the bacterium; cross-resistance between macrolides and lincosamides occurs more commonly than lincosamide resistance alone [40]. This multi-resistance can be constitutive where bacteria show high MIC values to both drugs or show dissociated inducible cross-resistance. In this case, bacteria initially resistant to macrolides can only develop resistance to lincosamides at the time they are exposed to macrolides. We did not investigate the presence of these mechanisms, but it is worth reporting that Italian *M. bovis* isolates also showed MIC90 values of >32 µg/mL, indicating a similar fate for both *Mycoplasma* species exposed to these compounds in the bovine respiratory airways. 

## 4. Material and Methods:

### 4.1. Mycoplasma Dispar Isolates Collection

A total of 41 *M. dispar* isolates was used in this study. The isolates derived from field or necropsy activities and were obtained by collecting nasal swabs from animals showing clinical signs or swabs from carcasses with gross pathology findings consistent with BRD. In detail, *M. dispar* was recovered from 9 nasal swabs (22%), 24 bronchoalveolar lavages (58.5%), and 8 lungs of dead animals (19.5%). The isolates were collected for routine diagnosis between 2011 and 2019. Five isolates (12.1%) were collected in 2011, 2 (4.9%) in 2012, 10 (24.4%) in 2015, 10 (24.4%) in 2016, 10 (24.4%) in 2017, 2 (4.9%) in 2018 and 2 (4.9%) in 2019. The nasal swabs collected in the field and were rapidly inoculated in a transport medium were rapidly inoculated in a PPLO transport medium following manufacturer’s instructions (BD Difco^TM^, Worthing, UK) and cultured for *Mycoplasma* spp. The bronchoalveolar lavages were kept refrigerated and transferred to the laboratory within 24 h from collection. The lungs were sampled by separating the cranial-right lobe from the rest of the lung. After cauterizing the cut surface, a swab was inserted in the main bronchus and rubbed against its walls. The swab was then vigorously mixed into a selective medium (Mammal Mycoplasma Liquid Medium, Mycoplasma Experience^®^, Reigate, UK). 

### 4.2. M. dispar Isolates In Vitro Cultivation and Identification.

At the laboratory, the sample media were inoculated into a Mammal Mycoplasma Liquid Medium (Mycoplasma Experience^®^, Reigate, UK) and incubated at 37 ± 1 °C with 5% CO_2_ for at least two weeks. The bronchoalveolar lavages were centrifuged before being put into the liquid medium. The broths were examined every day and were inoculated onto agar plates of Mammalian Mycoplasma Agar (Mycoplasma Experience^®^, Reigate, UK) when a change in colour (from orange to yellow) or turbidity was observed. If no change occurred after 14 days, a blind passage on solid medium of Mammal Mycoplasma Experience^®^ was performed and plates were checked daily for the presence of any “fried-egg-like” colony. The samples were considered negative if no colony was observed during the following 7 days. For *Mycoplasma* species identification, DNA extraction was performed using an aliquot of 300 µL from positive suspect broths. The Maxwell^®^ 16 Blood DNA Purification Kit (Promega Italia Srl, Milano, IT) was used for DNA extraction. After DNA extraction, 16S-rDNA PCR and denaturing gradient gel electrophoresis (DGGE) were performed as described [41].

### 4.3. MIC Test for Mycoplasma dispar

MIC analysis was performed using a modified broth microdilution method based on the guidelines of Hannan [26] and a standardized procedure used for human mycoplasmas [42]. Each *M. dispar* isolate was passaged in vitro three times, first in liquid and then on solid media to obtain pure cutures [43]. At the third passage in liquid medium, the *M. dispar* suspension was transferred to 10 mL of Mycoplasma Experience^®^ (ME, Reigate, UK) without inhibitors. This stock solution held at the exponential growth phase was divided into five sub-aliquots, each one containing 1 mL then stored at -80°C for at least 24 h. The following day, an aliquot was thawed and titrated in 96-well microtitre plates to calculate the colour changing units (CCU/mL) using the most probable number method [43]. In addition, each isolate was tested again by DGGE before performing the MIC test to confirm its purity and identity. In order to achieve a standard final inoculum in each well, frozen sub-aliquots were thawed, diluted and adjusted to contain approximately 10^4^ CCU/mL in liquid medium without inhibitors. Custom-made 96-well microtitre plates were purchased containing lyophilized antimicrobials (Merlin Diagnostik^®^, LOT ES-295-100 140919P95001) comprising enrofloxacin, oxytetracycline, doxycycline, erythromycin, tylosin, tilmicosin, spiramycin, tiamulin, florfenicol and lincomycin. The range of antibiotic concentrations expressed in µg/mL are reported in Table 1. On each plate, one well on each plate contained only liquid medium alone and served as a negative control.

Plates were incubated aerobically at 37 ± 1 °C and were examined within 24–48 hours after inoculation when the positive-control-well showed typical signs of mycoplasma growth. Each isolate was tested in duplicate and results were considered valid when both MIC tests produced the same results. Moreover, the *M. dispar* reference strain (*M. dispar* 462/2, NCTC 10125) was added as a control strain at each work session. 

The MIC value of the antimicrobials was considered to be the lowest concentration that completely inhibited the growth or metabolism of the isolate. The MIC value was expressed as greater than (>) the highest antibiotic concentration present in the plate when growth was not inhibited by the highest antimicrobial concentration. In contrast, if growth was inhibited by the lowest antibiotic concentration present in the plate, the MIC value was expressed as lower than or equal (≤) to this concentration. MIC50 and MIC90, namely the lowest concentration of an antimicrobial capable to inhibit 50% and 90% of bacterial isolates respectively, were calculated. 

### 4.4. Statistical Analyses

The statistical analyses were conducted under R environment [44]. MIC value class frequency variation over the years was analyzed by asymptotic linear-by-linear association test (package “coin” [45]), a chi-square test implemented to assess linear relationships between ordinal variables (in this case “year” and “MIC value class”). This test is more powerful than Pearson chi-square when considering variables whose classes can be ranked according to their magnitude. The statistical relation between the enrofloxacin MIC class frequency and time was further analyzed via a proportional odds model (package “ordinal” [46]), using a logit link function and including “time” as independent variable. This latter was designated as a numeric vector ranging from 0 to 8 by mathematically centering the year of isolation on 2011. The proportional odds is a special case of ordinal model which assumes that the frequency of observation of each MIC value class follows a cumulative distribution (the cumulative probability of observing a certain MIC value class derives from the sum of the probabilities of the class itself and of all the lower classes). In addition, the employed model assumes that the probability of a time-dependent class change event is the same for all classes. Such assumption was verified with the function *scale_test* implemented in the same package (“ordinal”), which assesses the goodness of the proportional odds assumption. 

## 5. Conclusions

In conclusion our results show that a drift towards high antimicrobial concentrations is occurring within the *M. dispar* population very similar to that seen with *M. bovis* [24,34]. The mechanisms behind antimicrobial resistance in *M. dispar* and *M. bovis* warrant investigation, since these two species seem to respond similarly when treated with the same drugs. It becomes increasingly important to continue studying MIC values in order to help veterinarians to treat cattle effectively, reducing the negative impact of BRD and to combat antibiotic resistance. 

## Figures and Tables

**Table 1:** Distribution of MIC values of the tested antimicrobials against the 41 Italian *M dispar* isolates collected from cattle between 2011–2019.

Antimicrobial	MIC Values (µg/mL)													
	0.0078125	0.015625	0.03125	0.0625	0.125	0.25	0.5	1	2	4	8	16	32	64
Enrofloxacin						5	2	3	5	2	11 ^50^	11 ^90^	2 *	
Oxytetracycline							10	6	5 ^50^	5	3	5	1 ^90^	6
Doxycycline					7	4	4	18 ^50^	3	2 ^90^	2	1		
Erythromycin							1			1		39 ^50-90^		
Tylosin			2	3	4	16 ^50^	5	3					3	5 ^90^
Tilmicosin								1	2	2	6	7	12 ^50^	11 ^90^
Spiramycin							12	8	11 ^50^		2	1	7 ^90^	
Tiamulin			1	1	2	5	19 ^50^	7	6 ^90^					
Florfenicol							32 ^50^	9 ^90^						
Lincomycin							17	14 ^50^	2			1		7 ^90^

The MIC values are expressed in μg/mL. The dilution range of each antibiotic is represented by the grey cells (e.g., enrofloxacin was tested from 0.125 to 16 µg/mL). Superscript numbers indicate the MIC50 and MIC90 values. * isolate numbers outside dilution range were not inhibited by the highest concentration of antibiotic; ^50^ MIC50; ^90^ MIC90.

**Table 2:** Results of the asymptotic linear-by-linear association test of antibiotic MIC value class frequencies versus years.

Antibiotic	*Z*-Value	*p*-Value
Doxicycline	+0.79	0.428
Enrofloxacin	+2.39	0.0168
Erythromycin	−1.68	0.0925
Florfenicol	+0.14	0.886
Lyncomycin	-1.68	0.0928
Oxytetracycline	+0.39	0.695
Spiramycin	−1.84	0.0654
Tiamulin	−2.06	0.0389
Tylosin	−2.21	0.0273
Tilmicosin	−1.43	0.152
Tylvalosin	+0.56	0.574

The Z-value is the test statistic used to support or reject the null hypothesis (random distribution of the MIC values throughout the years). Its sign (+/−) indicates the presence of a either a positive or a negative correlation between the ordinal variables.

**Table 3:** Parameter estimates with relative standard error (Std. Error), Wald statistic (*Z*-value) and *p*-value of the proportional odds model relating the frequency of observation of the different enrofloxacin MIC value classes to the variable “year”.

Coefficients	Estimate	Std. Error	*Z*-Value	*p*-Value
Time	0.331	0.136	2.44	0.0147
**Threshold coefficients**				
0.25|0.5	−0.691	0.689	−3.056	
0.5|1	−0.257	0.663	−0.388	
1|2	0.256	0.662	0.391	
2|4	0.933	0.683	1.367	
4|8	1.151	0.686	1.677	
8|16	2.319	0.725	3.198	
16|>16	4.591	0.992	4.629	
